# Wrapping gastroduodenal artery stump with the teres hepatis ligament to prevent postpancreatectomy hemorrhage after pancreaticoduodenectomy

**DOI:** 10.1186/s12957-023-03218-z

**Published:** 2023-11-27

**Authors:** Xiang Zheng, Zedong Jiang, Zhenzhen Gao, Bo Zhou, Guogang Li, Sheng Yan, Xiaoping Mei

**Affiliations:** 1 Department of Hepatobiliary and Pancreatic Surgery, The Second Affiliated Hospital Zhejiang University School of Medicine, Hangzhou, China; 2 Key Laboratory of Precision Diagnosis and Treatment for Hepatobiliary and Pancreatic Tumor of Zhejiang Province, Hangzhou, China; 3 Affiliated Hospital of Jiaxing University (The First Hospital of Jiaxing), Jiaxing, China

**Keywords:** Pancreaticoduodenectomy, Gastroduodenal artery, Postpancreatectomy hemorrhage, Pancreatic fistula, Teres hepatis ligament

## Abstract

**Background:**

Gastroduodenal artery (GDA) stump erosion hemorrhage is a fatal complication after pancreaticoduodenectomy. This study aimed to determine whether GDA stump wrapping with the teres hepatis ligament during pancreaticoduodenectomy decreased the incidence of postpancreatectomy hemorrhage (PPH).

**Methods:**

We reviewed 307 patients who had undergone pancreaticoduodenectomy between March 2019 and June 2022. The patients were divided into two groups according to application of GDA stump wrapping with the teres hepatis ligament: GDA wrapping group (165 patients) and no-wrapping group (142 patients). The perioperative data were compared between the groups.

**Results:**

The clinical characteristics were balanced between the two groups. Grades B and C PPH and GDA-stump-related hemorrhage were significantly reduced in the GDA wrapping group compared with the no-wrapping group (PPH B/C, 13.4% vs 6.1%, *P* = 0.029; GDA hemorrhage, 5.6% vs 0.6%, *P* = 0.014). No difference was observed in the incidence of clinically relevant postoperative pancreatic fistula, biliary leak, intra-abdominal abscess, delayed gastric emptying, 90-day mortality, and postoperative hospital stay between the two groups.

**Conclusion:**

Wrapping GDA stump with the teres hepatis ligament reduced the incidence of GDA-stump-related PPH. Therefore, the wrapping technique is a simple and effective strategy to prevent PPH. Prospective studies are needed to confirm the benefit of this procedure.

## Introduction

Pancreaticoduodenectomy (PD) is a standard surgical procedure for pancreatic head and periampullary carcinomas. This procedure is associated with high morbidity and mortality [1, 2] Postoperative pancreatic fistula (POPF) and postpancreatectomy hemorrhage (PPH) are the two major complications. Although POPF is not a direct cause of death, PPH can be fatal. PPH rate after PD occurs in 3–16% of patients, and the gastroduodenal artery (GDA) stump is a frequent site of bleeding [3–7]. Possible pathophysiological explanations for GDA-stump-related PPH include erosion and pseudoaneurysm formation of the GDA stump by pancreatic juice or local infection secondary to POPF [8].

Several methods have been developed to prevent POPF and GDA-stump-related PPH. Wrapping pancreatoenteric anastomosis or skeletal vessels with omental flaps or ligaments is one of the procedures to protect the surrounding organs against pancreatic juice [9–12]. However, previous studies have shown that this surgical technique may not reduce the incidence of POPF [13]. The protective effect of the vessel wrapping procedure against GDA-stump-related PPH is under debate [14]. Therefore, we performed this study to evaluate whether GDA wrapping using the teres hepatis ligament helped to prevent PPH after PD.

## Materials and methods

### Patients

Between March 2019 and June 2022, the medical records of patients who underwent PD at the Second Affiliated Hospital of Zhejiang University and Affiliated Hospital of Jiaxing University were reviewed for eligibility. The exclusion criteria were no teres hepatis ligament available to create a wrapping or no creation of a pancreaticoenterostomy. Patients were divided into two groups depending on whether they had wrapping of the GDA stump by the teres hepatis ligament. It is each surgeon’s preference to or not to do the wrapping. All clinical, biochemical, and radiological data were retrospectively collected from the database. The study protocol was approved by the Ethics Committee of the Second Affiliated Hospital of Zhejiang University (No: 2023–0667). All patients provided written informed consent before inclusion.

### Perioperative morbidity

Perioperative morbidity included PPH, POPF, biliary leak, delayed gastric emptying, wound infection, intra-abdominal abscess, pneumonia, and heart failure. PPH, POPF, and delayed gastric emptying were defined and graded according to the international consensus definitions of the International Study Group of Pancreatic Surgery (ISGPS) [3, 15, 16].

### GDA stump wrapping procedure

**Fig. 1:**
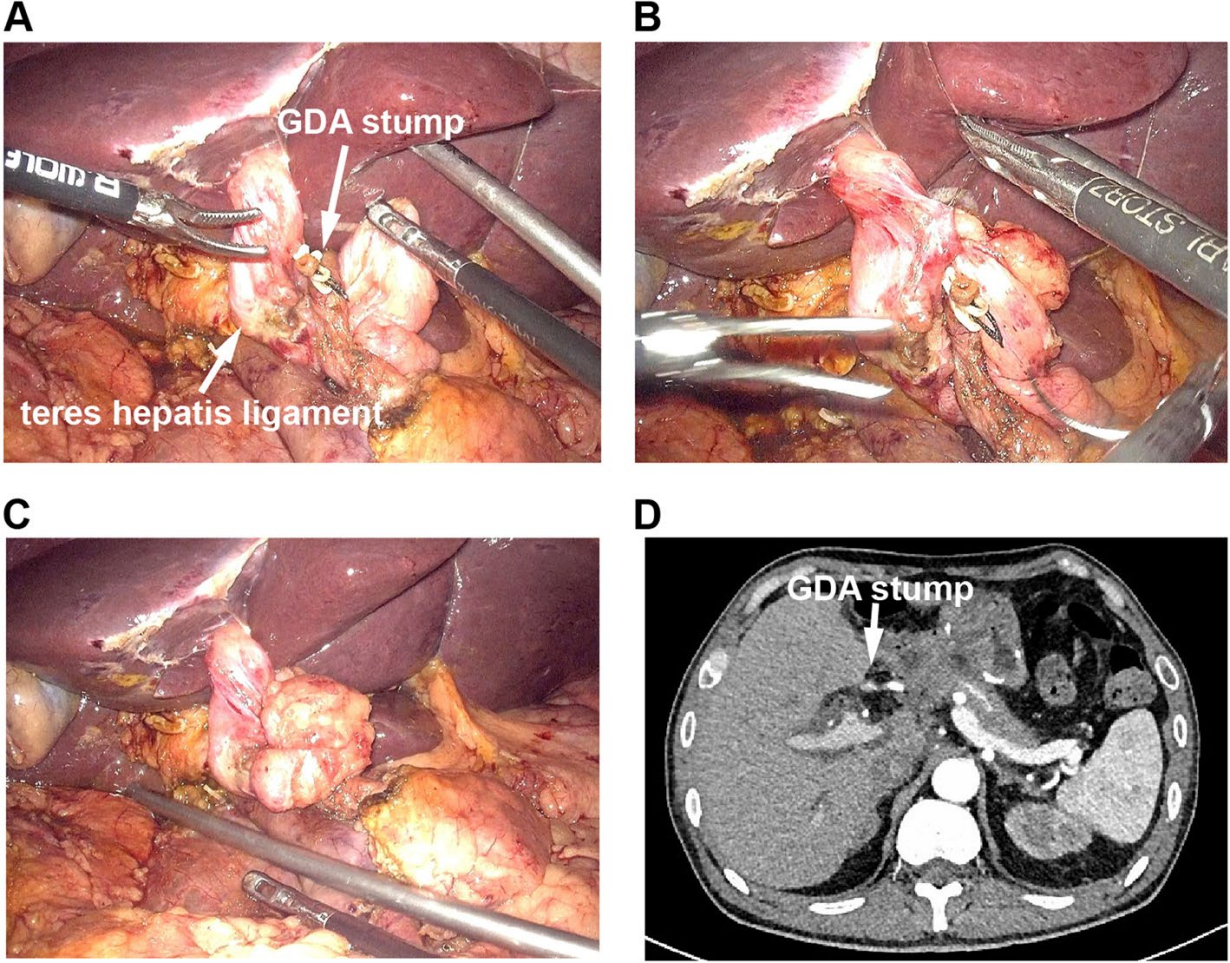
The prepared pedicled teres hepatis ligament was wrapped around the divided GDA stump. **A** The prepared pedicled teres hepatis ligament was placed below the GDA stump. **B** The teres hepatis ligament was used to wrap the GDA stump. **C** The GDA stump was completely covered by the teres hepatis ligament. **D** Postoperative enhanced computed tomography scan showed the wrapped GDA stump (arrow) was fully covered by the teres hepatis ligament (fat density)

Division of the GDA was routinely performed using 4–0 polypropylene sutures or two hem-o-lok clips. After the completion of PD, the teres hepatis ligament was mobilized after division of the falciform ligament close to the umbilicus. The teres hepatis ligament was separated from the liver parenchyma to ensure achievement of a ligament length of 10–15 cm. The blood supply and fat near the ligament were preserved. The prepared pedicled teres hepatis ligament was placed below the divided GDA stump and wrapped around it in a tension-free manner (Fig. 1A–C). Postoperative contrast-enhanced computed tomography was routinely performed to confirm that the GDA stump was completely covered by the teres hepatis ligament (Fig. 1D).

### Statistical analysis

Continuous variables were expressed as mean ± standard deviation, and between-group differences were compared using Student’s *t*-test. Categorical variables were compared using Pearson’s *χ*^2^-test or Fisher’s exact test. Two-tailed *P* < 0.05 was considered statistically significant. All statistical analyses were performed using SPSS 26.0 software.

## Results

### Patient characteristics

Between March 2019 and June 2022, 307 patients who underwent PD were categorized into two groups: 142 without (no-wrapping group) and 165 with (GDA wrapping group) wrapping of GDA stumps (Table 1). The median age of the total cohort was 64.0 (interquartile range 57.0–71.0) years. The main primary diseases were pancreatic ductal adenocarcinoma (61.2%) and periampullary carcinoma (28.3%). Preoperative biliary drainage was performed in 84 (27.4%) patients, and 65 (21.2%) underwent neoadjuvant treatment prior to surgery. Both open (30.7%) and minimally invasive (69.3%) PD were included in this study. The two groups were well balanced regarding demographics, health status, and disease characteristics.

**Table 1:** Patient characteristics

	No wrapping (*n* = 142)	GDA wrapping (*n* = 165)	*p*-value
Gender			0.867
Male	83 (58.5%)	98 (59.4%)	
Female	59 (41.5%)	67 (40.6%)	
Age (years, mean ± SD)	63.3 ± 10.6	63.8 ± 9.4	0.710
BMI (kg/m^2^, mean ± SD)	22.1 ± 2.9	22.3 ± 2.9	0.536
Smoking	42 (29.6%)	55 (33.3%)	0.480
Hypertension	38 (26.8%)	36 (21.8%)	0.313
Diabetes mellitus	24 (16.9%)	22 (13.3%)	0.382
Preoperative total bilirubin (mg/dL, mean ± SD)	68.9 ± 85.2	59.6 ± 76.0	0.315
Preoperative biliary drainage	41 (28.9%)	43 (26.1%)	0.582
Open/minimally invasive PD	58/84 (40.8%/59.2%)	71/94 (43.0%/57.0%)	0.699
Neoadjuvant treatment	25 (17.6%)	40 (24.2%)	0.156
Pathology			0.745
PDAC, pancreatic ductal adenocarcinoma	86 (60.6%)	102 (61.8%)	
IPMN, intraductal papillary mucinous neoplasm	8 (5.6%)	9 (5.5%)	
Distal cholangiocarcinoma/ampullary carcinoma	39 (27.5%)	48 (29.1%)	
Other	9 (6.3%)	6 (3.6%)	

*BMI* body mass index, *PDAC* pancreatic ductal adenocarcinoma, *IPMN* intraductal papillary mucinous neoplasm

### Perioperative characteristics

The perioperative characteristics are listed in Table 2. The operating time did not differ significantly between the GDA wrapping (364 ± 113 min) and no-wrapping (368 ± 106 min) groups. The mean tumor size was 2.62 ± 1.34 cm. A combined portal vein/superior mesenteric vein resection was performed in 27 patients (8.8%). The diameter of the Wirsung duct was ≤ 3 mm in 218 (71.0%) patients, and the pancreatic parenchyma had soft texture in 192 (62.5%) patients. All pancreaticoenterostomies were performed with the jejunum, and 317 (94.3%) patients underwent pancreaticoenterostomy via a duct-to-mucosal anastomosis. There were no significant differences between the two groups for any of the perioperative characteristics.

**Table 2:** Perioperative characteristics

	No wrapping (*n* = 142)	GDA wrapping (*n* = 165)	*p*-value
Venous resection	11 (7.7%)	16 (9.7%)	0.547
Perioperative blood transfused	25 (17.6%)	24 (14.5%)	0.465
Operating time (min)	368 ± 106	364 ± 113	0.755
Estimate blood loss (mL, mean ± SD)	232 ± 170	216 ± 193	0.441
Pancreatic texture (*n*, %)			0.778
Soft	90 (63.4%)	102 (61.8%)	
Firm	52 (36.6%)	63 (38.2%)	
Pancreatic duct diameter (*n*, %)			0.223
≤ 3 mm	96 (67.6%)	122 (73.9%)	
> 3 mm	46 (32.4%)	43 (26.1%)	
Pancreaticoenterostomy			
Jejunum/stomach	142/0	165/0	
Duct-to-mucosal anastomosis (yes/no)	136/6 (95.8%/4.2%)	156/9 (94.5%/5.5%)	0.618
Usage of pancreatic stent tube (yes/no)	135/7 (95.1%/4.9%)	158/7 (95.8%/4.2%)	0.774
Tumor size (cm, mean ± SD)	2.7 ± 1.3	2.5 ± 1.4	0.185

### Postoperative morbidity

The postoperative complications are listed in Table 3. Clinically relevant PPH, defined according to the ISGPS criteria, was identified in 19 (13.4%) patients in the no-wrapping group and 10 (6.1%) in the GDA wrapping group (*P* = 0.029). Eight patients (5.6%) developed GDA-stump-related PPH in the no-wrapping group, compared with only one patient (0.6%) in the GDA wrapping group (*P* = 0.014). The anatomical location of the main sites of bleeding in patients with clinically relevant PPH is listed in Table 4. Fourteen patients (73.7%) with grade B/C PPH in the no-wrapping group and five (50.0%) in the GDA wrapping group experienced hemorrhage from an artery. The most frequent anatomical sites of PPH included the GDA stump, proper hepatic artery, common hepatic artery, and gastrojejunostomy. Clinically relevant POPF was seen in 19 (13.4%) patients in the no-wrapping group and 26 (15.8%) patients in the GDA wrapping group (*P* = 0.557). Other complications included biliary leak, delayed gastric emptying, intra-abdominal abscess, pneumonia, and heart failure, and these did not differ significantly between the two groups. No differences were observed in 90-day reoperation and mortality between the two groups. The mean postoperative hospital stay was 28.0 ± 10.6 and 30.2 ± 17.3 days in the no-wrapping and GDA wrapping groups, respectively (*P* = 0.174).

**Table 3:** Postoperative morbidity

	No-wrapping (*n* = 142)	GDA wrapping (*n* = 165)	*p*-value
PPH grade B/C	19 (13.4%)	10 (6.1%)	0.029
GDA hemorrhage	8 (5.6%)	1 (0.6%)	0.014
POPF grade B/C	19 (13.4%)	26 (15.8%)	0.557
Biliary leak	5 (3.5%)	7 (4.2%)	0.745
Delayed gastric emptying	6 (4.2%)	9 (5.5%)	0.618
Intra-abdominal abscess	10 (7.0%)	14 (8.5%)	0.639
Pneumonia	7 (4.9%)	5 (3.0%)	0.392
Heart failure	0	1 (0.6%)	1.000
90-day reoperation	7 (4.9%)	4 (2.4%)	0.239
90-day mortality	4 (2.8%)	2 (1.2%)	0.421
Postoperative hospital stay (days)	28.0 ± 10.6	30.2 ± 17.3	0.174

**Table 4:** Anatomical location of main bleeding in patients with grade B/C PPH

Anatomical location	No wrapping (*n* = 19)	Wrapping (*n* = 10)
GDA	8	1
PHA/CHA	4	2
Left gastric artery	0	1
SMA branch	2	1
PV/SMV	1	1
Pancreatic anastomosis	1	1
Gastric ulcer/gastrojejunostomy	2	3
Unknown	1	0

*PHA* proper hepatic artery, *CHA* common hepatic artery, *SMA* superior mesenteric artery, *PV* portal vein, *SMV* superior mesenteric vein

## Discussion

PPH remains one of the major complications after PD. However, it carries a high mortality of ~ 20% [6, 24]. PPH is differentiated by the ISGPS into early (≤ 24 h after the end of the index operation) and late (> 24 h) based on the time of onset [3]. Early PPH mainly occurs after technical failure of appropriate hemostasis during the operation, while late PPH usually occurs several days or even weeks after the operation and is usually related to surgical complications. The majority of late PPH arises from an eroded or ruptured splanchnic artery secondary to POPF and/or intra-abdominal infection, and the GDA stump is one of the most frequent sources of late PPH [25–28]. In this study, the GDA stump accounted for 42% (8/19) of the PPH in the no-wrapping group, which agrees with previous studies [29, 30]. Thus, prevention of POPF and GDA-stump-related PPH is major concerns in PD.

The wrapping technique using the omental flap and ligament was developed to protect the skeletonized vessels and pancreatic enteric anastomosis [10–12, 31–33]. Wrapping the omental flap and ligament around the pancreatic anastomosis was a method to reinforce the pancreaticojejunostomy, as the omentum and ligament provided a source of granulation tissue and neovascularization to promote healing [14, 17, 18, 21, 34]. Others chose to protect the exposed major blood vessels from pancreatic juice digestion by wrapping them with the omental flap and ligament, as they believed that pancreatic leak could not be avoided completely, but vessel erosion hemorrhage was life-threatening. Several studies have shown that the wrapping does not markedly decrease the incidence of POPF but protects the splanchnic vessels from erosion hemorrhage [19, 22, 23, 35, 36]. However, other studies have shown different conclusions [13, 37]. A retrospective study of the Japanese Society of Pancreatic Surgery indicated that using omental flap or falciform ligament neither decreased the occurrence of POPF nor PPH after PD [13]. To date, no consensus has been reached on these methods to reduce the incidence of POPF and PPH (Table 5).

**Table 5:** Studies (cases ≥ 50) comparing PD with and without wrapping in the English language literature

Year	Authors	Study design	Study type	Inclusion period	Country	Patients enrolled (*n*)		Wrapping location	Wrapping material	PPH (wrapping vs. no wrapping)	Conclusion
2012	Tani et al. [13]	Retrospective observational survey	Multicentric	2006–2008	Japan	2597	W 918	Pancreatic anastomosis or vessels (not specified)	Omentum or falciform ligament	Overall intra-abdominal hemorrhage, 3.2% vs. 3.2%	No difference in intra-abdominal hemorrhage
							nW 1679				
2012	Rosso et al. [17]	Retrospective observational	Monocentric	2009–2009	France	61	W 33	Pancreatic anastomosis, hepatic artery and celiac trunk	Omentum	Overall PPH, 3.0% vs. 10.7%	No difference in PPH
							nW 28				
2012	Choi et al. [18]	Retrospective observational	Monocentric	2009–2011	Korea	68	W 29	Pancreaticojejunostomy	Omentum	Overall PPH, 6.9% vs. 7.7%	Reduce the incidence of POPF but not PPH
							nW 39				
2014	Xu et al. [19]	Retrospective observational	Monocentric	2005–2012	China	280	W140	GDA stump	Teres hepatis ligament	GDA stump-related PPH, 0.7% vs. 6.4%	Reduce the postoperative GDA-related hemorrhage incidence
							nW140				
2016	Kapoor et al. [20]	Retrospective observational	Monocentric	1989–2015	India	784	W 132	GDA stump and pancreaticojejunostomy	Omentum	Overall PPH, 14% vs. 18%	No difference in overall PPH, less delayed intra-abdominal bleeds in wrapping group
							nW 652				
2017	Müssle et al. [12]	Retrospective observational	Monocentric	2012–2015	Germany	196	W 39	GDA stump	Falciform ligament	GDA stump-related PPH, 7.7% vs. 9.6%	The incidence of erosion hemorrhage after wrapping is low, but there are still insufficient controlled data to support its general use
							nW 157				
2021	Tangtawee et al. [21]	Randomized Clinical Trial	Monocentric	2017–2019	Thailand	68	W 34	Pancreaticojejunostomy	Omentum	Overall PPH, 2.9% vs. 2.9%	Neither reduce PPH nor POPF
							nW 34				
2021	Meng et al. [22]	Retrospective observational	Monocentric	2016–2019	China	247	W 119	GDA stump	Teres hepatis ligament	Overall PPH B/C, 0% vs. 5.5%	Reduce the rate of PPH of Grade B and C
							nW 128				
2022	Welsch et al. [23]	Randomized Clinical Trial	Multicentric	2015–2020	Germany	417	W 207	GDA stump	Falciform ligament	GDA stump-related PPH, 2.9% vs. 7.1%	Lower the rate of bleeding from the hepatic artery or GDA stump
							nW 210				
Present study	Zheng et al	Retrospective observational	Multicentric	2019–2022	China	307	W 165	GDA stump	Teres hepatis ligament	Overall PPH B/C, 13.4% vs. 6.1%; GDA stump-related PPH, 5.6% vs. 0.6%	Reduce the incidence of GDA stump-related PPH
							nW 142				

*W* wrapping, *nW* no wrapping

The wrapping technique was simple to perform either in open or minimally invasive surgery. The wrapping procedure to mobilize the teres hepatis ligament and wrap the GDA stump took an average of 5–10 min; thus, this step did not overly prolong operating time (Table 2). The common wrapping materials included omental flap, falciform ligament, and teres hepatis ligament. We chose to wrap the GDA stump with the teres hepatis ligament rather than the omental flap or falciform ligament for the following reasons. Firstly, the location of the teres hepatis ligament in the porta hepatis made it easy to divide and harvest in open or laparoscopic surgery. Secondly, the structure of the teres hepatis ligament covered by peritoneum was thicker and stronger than the omentum or falciform ligament. Thirdly, wrapping associated complications, like panniculitis, intra-abdominal infection, intestinal obstruction, and flap necrosis, have been reported in previous studies with omental flap [10, 11].

In the present study, we included a no-wrapping group as a control. The overall incidence of grade B/C PPH and clinically relevant POPF in the control group was 13.4% and 13.4%, respectively, which corresponded with previous studies in high-volume centers [22, 23]. The overall incidence of grade B/C PPH and clinically relevant POPF was 6.1% and 15.8% in the wrapping group, respectively. Our study revealed that the GDA stump wrapping significantly decreased the overall incidence of grade B/C PPH (13.4% vs 6.1%, *P* = 0.029) and GDA-stump-related hemorrhage (5.6% vs 0.6%, *P* = 0.014), indicating the vessel-protective effect of wrapping. However, no difference in POPF was found between the two groups, indicating that the GDA stump wrapping did not lower the incidence of POPF. Wrapping had no obvious influence on biliary leak and delayed gastric emptying, and wrapping-associated complications reported in previous studies, such as intra-abdominal infection hepatic artery stenosis, did not increase in the wrapping group compared with the no-wrapping group. Despite the high mortality rate in cases of PPH, the lower rate of PPH in the wrapping group did not translate into a significantly lower mortality rate (2.8% vs 1.2%, *P* = 0.421). This might have been due to the overall low rate of GDA-stump-related PPH. The application of interventional angiography and transcatheter arterial embolization for arterial hemorrhage also rescued most of the PPH. Eighty percent of the patients (4/5) in the wrapping group and 71.4% (10/14) in the no-wrapping group had successful hemostasis by transcatheter arterial embolization (data not shown). Thus, the mortality caused by PPH was too low to affect the statistical results.

This was a multicenter retrospective study with a small sample size, which may have led to selection bias. Therefore, well-designed randomized controlled trials are needed to verify the benefits of this technique in PD.

## Conclusion

In conclusion, our study showed that wrapping the GDA stump with the teres hepatis ligamentum decreased the incidence of GDA-stump-related hemorrhage and grade B/C PPH. Such a wrapping procedure, which is simple to perform in open or minimally invasive surgery, without increasing the operating time and other complications, can protect the GDA stump from pancreatic juice and prevent hemorrhage.

## Data Availability

The datasets used and/or analyzed during the current study are available from the corresponding author upon reasonable request.

## Abbreviations

GDA: Gastroduodenal artery
PPH: Postpancreatectomy hemorrhage
PD: Pancreaticoduodenectomy
POPF: Postoperative pancreatic fistula
ISGPS: International Study Group of Pancreatic Surgery
